# Feasibility and impact of a monoclonal antibody infusion program in reaching vulnerable underserved communities

**DOI:** 10.1017/ice.2023.25

**Published:** 2023-10

**Authors:** Alfredo J. Mena Lora, Stephanie L. Echeverria, Brenna Lindsey, Ella Li, Lawrence Sanchez, Tiffany Truesdell, Eden Takhsh, Romeen Lavani, Rodrigo Burgos

**Affiliations:** 1 University of Illinois at Chicago, Chicago, Illinois; 2 Saint Anthony Hospital, Chicago, Illinois; 3 Arizona College of Osteopathic Medicine, Midwestern University, Glendale, Arizona

## Abstract

The coronavirus disease 2019 (COVID-19) pandemic has disproportionately impacted Black, indigenous, and people of color (BIPOC). Equitable access to therapeutics is key to addressing health disparities. We established a monoclonal infusion program in the emergency department of a safety-net hospital. Our program successfully reached underserved BIPOC communities and was sustained throughout the pandemic.

The coronavirus disease 2019 (COVID-19) pandemic has resulted in significant morbidity and mortality worldwide. Its disproportionate impact on vulnerable minority populations was apparent early, shedding light on enduring healthcare inequities. Black, indigenous, and people of color (BIPOC) experienced increased rates of infection, hospitalization, and mortality compared to non-BIPOC persons.^1^ Addressing disparities with equitable access to vaccines and therapeutics is essential. Monoclonal antibodies (mAbs) against COVID-19 are potent therapeutics that neutralize the spike protein of severe acute respiratory coronavirus virus 2 (SARS-CoV-2) to prevent viral entry and limit disease progression in high-risk patients.^2–4^ The US Food and Drug Administration (FDA) granted emergency use authorization (EUA) to the first anti–SARS-CoV-2 mAb in November 2020, coinciding with the COVID-19 mass vaccination campaign and a COVID-19 surge. Staffing shortages and logistical issues in an already overburdened healthcare system made operationalizing mAb infusions challenging. Furthermore, existing healthcare disparities and barriers to access may have reduced uptake of therapeutics in BIPOC communities where they were needed most. Barriers include limited access to prompt COVID-19 testing, transportation, inadequate insurance, language, reduced availability of mAb infusion locations, and reduced linkage to care. We established an mAb infusion program centered in the emergency department (ED) fast-track location at an urban safety-net community hospital. Herein, we have described our program and have assessed its feasibility and impact in reaching vulnerable underserved communities.

## Methods

### Study design

We conducted a retrospective study of deidentified data reported to the Chicago Department of Public Health (CDPH) on mAb utilization. Doses given at Saint Anthony Hospital from November 2020 to February 2022 were reviewed and categorized by ZIP code and ethnicity to determine utilization in BIPOC communities of Chicago. The COVID-19 community vulnerability index, a formula adapted by CDPH the CDC social vulnerability index (CCVI), identifies communities disproportionately affected by COVID-19 using sociodemographic, epidemiological, occupational factors and COVID-19 burden.^5^ Communities were defined by ZIP code as low, medium, or high vulnerability to COVID-19.^5^ The University of Illinois at Chicago Institutional Review Board approved this study.

### Study setting and mAb infusion process

Saint Anthony Hospital is a 151-bed urban safety-net community hospital located in the West Side of Chicago, Illinois, in a ZIP code with a high CCVI. The ED fast-track location was repurposed for mAb infusions. The fast-track location is an 8-bed area designated for ED visits of low complexity. Two beds were assigned for mAb infusions. Indications and protocols based on the FDA EUA were developed by the infectious disease (ID) physician and antimicrobial stewardship program (ASP). The protocol was modified as variants emerged and new agents were developed. A referral program connecting patients from telehealth and providers in the community to the infusion program was established along with a test-to-treat protocol in the ED. Times with lowest ED volume were set aside for mAb referrals. In the ED, patients with COVID-19–like symptoms would undergo immediate point-of-care COVID-19 PCR testing, and high-risk individuals would be referred to the mAb program if positive. Referrals from outside the ED were sent from providers in the community, our COVID-19 telehealth services, or employee health services. The community providers included Federally Qualified Health Centers (FQHCs) and private clinics. The referral process involved communicating via email with patient indications for mAbs, date of SARS-CoV-2 test and date of symptoms. ID and ASP staff reviewed and approved mAb use, and the ED coordinator scheduled a same-day or next-day infusion time. The program was staffed by ED nurses and physicians.

## Results

**Fig. 1.**
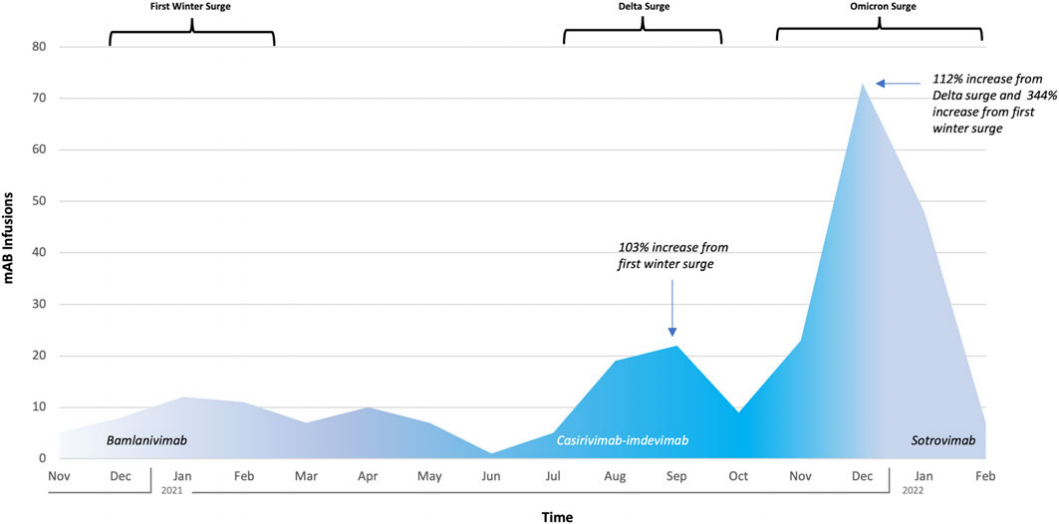
Monoclonal antibody **(**mAb) doses across COVID-19 surges.

**Fig. 2.**
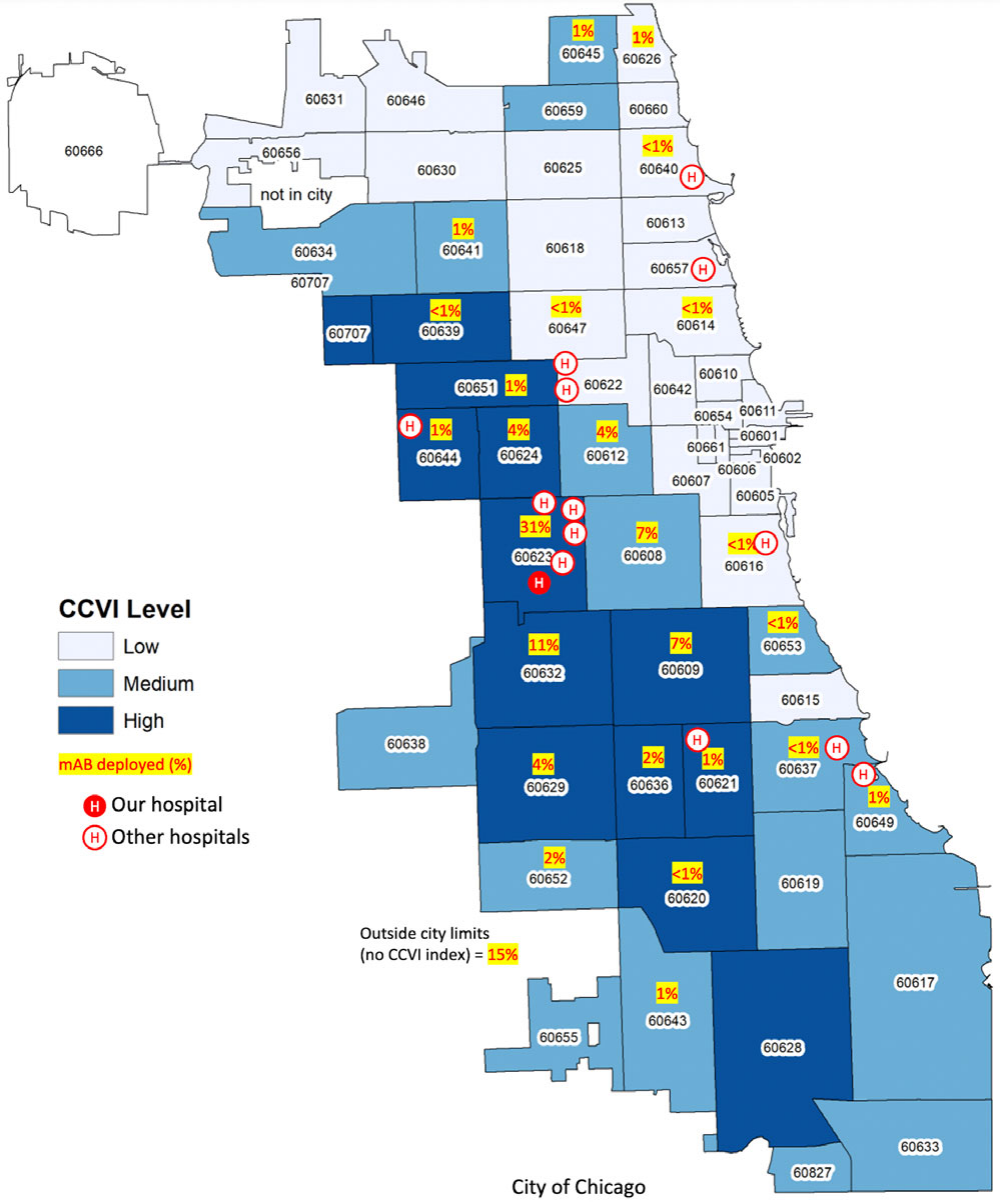
ZIP codes based on CDC social vulnerability index (CCVI) levels, monoclonal antibody **(**mAb) utilization, and location of our facility.

In total, 267 patients were treated within our study period, with an average age of 52 years, of whom 55% (147) were female. The ethnicity of those receiving mAb infusions was 46% Hispanic and race was 54% White, 28% Black, 1.4% Asian, 1.8% multiracial, and 14% other. White non-Hispanics received 15% of infusions. Utilization increased with each COVID-19 surge. A dose distribution increase of 103% occurred between the first winter surge and the surge of the SARS-CoV-2 δ (delta) variant, and a subsequent increase of 112% occurred between the SARS-CoV-2 δ (delta) variant surge and the SARS-CoV-2 ο (omicron) variant surge (Fig. 1). Utilization increased by 344% from the first winter surge to the SARS-CoV-2 ο (omicron) surge. The program was sustainable, and protocols were successfully adapted to novel mAbs as new COVID-19 variants emerged. ZIP codes were categorized: 64% had a high CCVI, 18% had a medium CCVI, and 3% had a low CCVI (Fig. 2).

## Discussion

A mAb infusion program with immediate test-to-treat pathway and a referral pathway for providers in underserved communities was successfully introduced and sustained in an ED fast-track location. Most patients served by our program were BIPOC (47% Hispanic, 28% Black), and the program primarily reached ZIP codes of high and medium CCVI risk (82%), reflecting the usual population served by our hospital. The ZIP codes in our program represented a wider reach than our usual population.

An analysis of nationwide mAb use revealed reduced utilization in BIPOC communities, with Hispanic patients receiving mAbs 58% less than non-Hispanics and Blacks, Asians, or others receiving mAbs 22%, 48%, and 47% less often, respectively, compared to Whites.^3^ A facility in our ZIP code reported that 78% of mAbs were used in BIPOC patients, comparable to our institution’s 85%.^6^ Thus, facilities located within BIPOC communities may be important in closing this gap, and existing relationships with these facilities, along with higher trust and accessibility, may play a role. Equitable access to medications and immunizations is important for current and future pandemics. Developing sustainable delivery models connecting medications with BIPOC communities is important to address these disparities. More than 70% of hospitals in the United States have <200 beds, and most safety-net hospitals serve areas in need and BIPOC communities.^7–9^ Thus, identifying models that are effective and sustainable in this setting is important to help reduce treatment gaps in BIPOC communities. Our program was sustainable in this setting and required no additional capital expenditures.

The ability to rapidly develop novel agents against emerging and re-emerging diseases make mAbs an alluring treatment.^2^ However, the COVID-19 pandemic shed light on important logistical challenges. Nationwide, mAb use was limited due to staffing shortages and space limitations amid COVID-19 surges and mass vaccination campaigns, leaving many doses unused at times of high need.^4^ Identifying delivery models that are sustainable in public health emergencies akin to COVID-19 is important. Our program leveraged existing space and staff in the ED to offset staffing and space challenges required for ambulatory infusion centers. Uptake increased with each wave, likely reflecting awareness of mAbs and our program by patients and providers in the community. The time-sensitive indication for mAbs poses another barrier, requiring administration within 7–10 days of symptom onset. Access to testing may pose a barrier in BIPOC communities, furthering healthcare disparities. Our program addressed this by establishing a test-to-treat pathway in the ED, facilitating referrals from the community, and providing same-day or next-day appointments for infusions.

Our mAb infusion program, led by ID and ASP staff, was successfully implemented in an ED fast-track location and sustained throughout the pandemic, reaching predominantly underserved BIPOC communities. By leveraging existing spaces and staff such as the ED fast-track setting, hospitals can establish mAb programs quickly without additional personnel or capital expenditures. Safety-net hospitals are often financially vulnerable, reflecting the payer mix of the communities they serve.^8,9^ Thus, finding cost-effective and sustainable ways to deliver therapeutics is of utmost importance in hospitals that serve BIPOC communities to reduce healthcare inequities for the current pandemic as well as inevitable future pandemics.
